# Supplementary material for: Identification of photoperiod-induced specific miRNAs in the adrenal glands of Sunite sheep (Ovis aries)
Source: Front Vet Sci. 2022 Jul 22;9:888207. doi: 10.3389/fvets.2022.888207 (PMC9354845; doi:10.3389/fvets.2022.888207)
Supplement: Supplementary file 1 [file Data_Sheet_1.ZIP › Supplementary file/miRDeep2 file.zip!/miRDeep2 file/Know_Structure/LP42A1/oar-mir-221.pdf]

miRBase precursor	: oar-mir-221
Total read count	: 2089
oar-miR-221 read count	: 1901
remaining reads	: 188

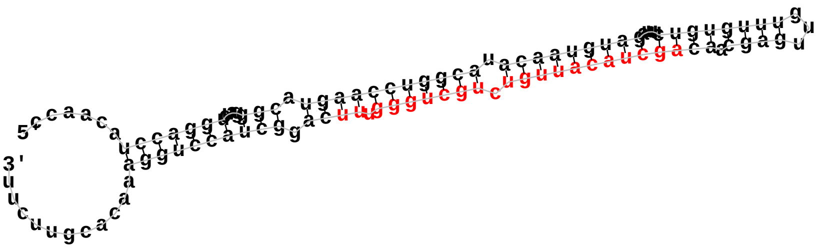

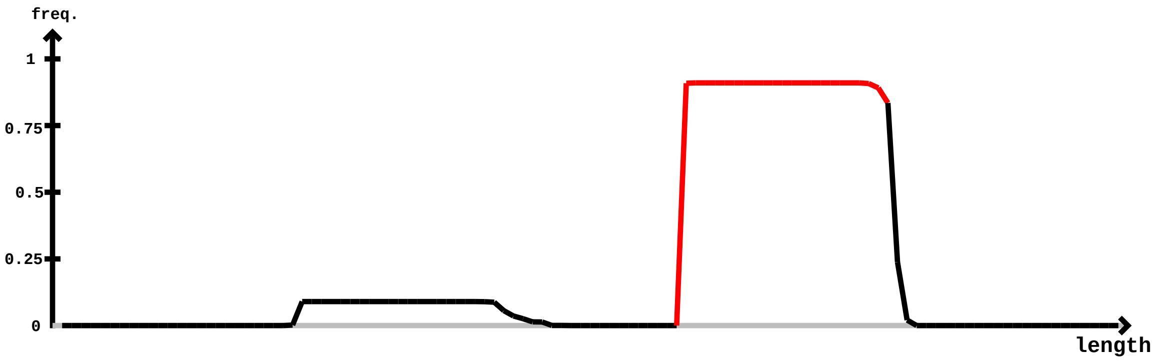

oar-miR-221

```
5'- ccaacauccaggucuagggcaugaaccuggcauacaauguagauuucuguguuuguugagcaacagcuacauugucugcuggguuucaggcuaccuggaaacacguucuu   -3'    exp
    ......((((((....(((.((((((((((.((((((((....((((((((...)))).))))))))))))).))))))).)))).))))))))))..........    reads      mm         sample
    .......................aaccuggcauacaauguagau..................................................................    1          0          seq
    .......................aaccuggcauacaauguagauu.................................................................    2          0          seq
    .......................aaccuggcauacaauguagauuu................................................................    1          0          seq
    ........................accuggcauacaauguaga...................................................................    1          0          seq
    ........................accuggcauacaauguagau..................................................................    3          0          seq
    ........................accuggcauacaauguagauu.................................................................    64         0          seq
    ........................accuggcauacaauguagauuu................................................................    41         0          seq
    ........................accuggcauacaauguagauuuc...............................................................    21         0          seq
    ........................accuggcauacaauguagauuucu..............................................................    25         0          seq
    ........................accuggcauacaauguagauuucug.............................................................    1          0          seq
    ........................accuggcauacaauguagauuucugu............................................................    27         0          seq
    ........................accuggcauacaauguagauuucugugu..........................................................    1          0          seq
    ................................................................agcuacauugucugcuggg...........................    5          0          seq
    ................................................................agcuacauugucugcugggu..........................    34         0          seq
    ................................................................agcuacauugucugcuggguu.........................    118        0          seq
    ................................................................agcuacauugucugcuggguuu........................    1246       0          seq
    ................................................................agcuacauugucugcuggguuuc.......................    452        0          seq
    ................................................................agcuacauugucugcuggguuuca......................    43         0          seq
    .................................................................gcuacauugucugcuggguuu........................    1          0          seq
    .................................................................gcuacauugucugcuggguuuc.......................    2          0          seq
```
